# OD27 Estimating The Causal Effect Of Adaptive Treatment Strategies Using Longitudinal Observational Data

**DOI:** 10.1017/S0266462324001582

**Published:** 2025-01-07

**Authors:** Yingying Zhang, Noemi Kreif, Alastair Bennett, Andrea Manca

## Abstract

**Introduction:**

Real-world data can help inform policymaking in health care by facilitating the evaluation of realistic treatment protocols. To generate robust evidence, analysts must address time-dependent confounders—variables influenced by past treatment decisions and affecting future treatment. Double-robust methods can help in eliminating bias by modeling both the treatment and the outcome mechanisms, using machine learning to improve model specification.

**Methods:**

Longitudinal targeted minimum loss-based estimation (LTMLE) is a double-robust method that handles time-varying confounding, currently with only a few applications on real-world data. We demonstrate the use of LTMLE to evaluate realistic treatment protocols by applying it on longitudinal registry data to compare various treatment protocols that involve the use of erythropoiesis-stimulating agents (ESA) for myelodysplastic syndromes patients. We define dynamic regimes that trigger initiating ESA when relevant criteria (e.g., low hemoglobin levels) are met and require continuing/stopping ESA based on the response to treatment. We estimate the effect of these protocols on survival and EuroQol 5-dimension questionnaire (EQ-5D) scores.

**Results:**

We study static treatment regimes where we compare patients always on treatment with patients always not on treatment, and we find the average effects of always administering ESA versus never administering it are positive but not significant on patients’ EQ-5D scores or on survival probabilities across all treatment time periods. We also study dynamic treatment regimes where decisions to initiate and continue/discontinue treatment over time depend on changing patient characteristics and responses to treatment. We find that patients following dynamic treatment regimes are predicted to score higher in EQ-5D and have longer survival probabilities than patients under static treatment regimes.

**Conclusions:**

The paper provides a tutorial and case study demonstration of the LTMLE model that can evaluate realistic treatment protocols using longitudinal observational data. It accounts for time-varying confounding in estimating treatment effects and can incorporate machine learning in improving accuracy of outcome prediction. The model has been applied in the setting of long follow-up times and gradually reduced sample size.

